# *Dientamoeba fragilis* Infection Mimicking Appendicitis Case Report: New Differential Diagnosis Considerations

**DOI:** 10.1177/00099228251375262

**Published:** 2025-09-28

**Authors:** Jason T. Tsichlis, Gaspar Rivera

**Affiliations:** 1 UCSF Benioff Children’s Hospital, San Francisco, CA, USA; 2 Zuckerberg San Francisco General Hospital, San Francisco, CA, USA; 3 Ann & Robert H. Lurie Children’s Hospital of Chicago, Chicago, IL, USA; 4 Department of Pediatrics, Zuckerberg San Francisco General Hospital, San Francisco, CA, USA

**Educational Objectives**

- Appendicitis is a common presentation in pediatric urgent cares, emergency departments, and primary clinic sites that can have various confounding presentations, and this case directs the reader’s attention to *Dientamoeba fragilis*, a parasite that can go overlooked when evaluating and working up acute abdominal pain.
- With the rise of global migration and the burgeoning discipline of newcomer medicine, the case report concludes that the role of expanded infectious workups with the consideration of stool ova and parasite testing for abdominal pain in these vulnerable populations is critical for providing high-value care.

## Introduction

Appendicitis is the most common pediatric surgical emergency, estimated to be the cause of roughly 10% of acute abdominal pain cases presenting to an emergency department.^1^ The condition is notoriously variable in presenting symptoms and as a result, has received sizeable investment in improved diagnostics, antibiotics, and surgical management to drastically reduce mortality and morbidity.^2^ In addition to a growing chorus of concern regarding parasitic causes of appendicitis,^3,4^ the rise in global migration with the emerging field of newcomer medicine in the United States has brought renewed attention to confounding presentations. Newcomers, who are children who immigrated less than 3 years prior, experience barriers to high quality health care and could be at greater risk for increased morbidity from unnecessary testing and delayed diagnosis.^5^ With an increasing understanding of parasitosis burden in recently immigrated children,^6^ there is a strong imperative to examine our current appendicitis differential diagnosis paradigms.

## Case Presentation

An 8-year-old female presented to urgent care with sharp, acute-on-chronic right lower-quadrant abdominal pain, which began the morning of presentation and persisted throughout the afternoon. Her mother described her as having difficulty ambulating due to the pain. They were unable to indicate whether the pain had migrated from the periumbilical region. She had abdominal pain that had been ongoing for the past 3 weeks with a waxing and waning presentation and had taken polyethylene glycol in the past for constipation. She denied nausea, vomiting, abdominal distention, fever, diarrhea, hematochezia, or melenic stools. She denied cough, congestion, or rhinorrhea. She did not have any unexplained pruritus. She also had no dysuria, urinary frequency, or urgency.

She had no other chronic conditions for which she took any medication and had no history of abdominal or inguinal hernias or previous surgeries. Her family history was notable for multiple close family members with a history of appendectomy, including her mother.

She and her mother recently arrived in the United States from rural Peru 3 months prior. Her workup upon arrival was negative for human immunodeficiency virus (HIV), hepatitis B, syphilis, and active tuberculosis. She had a recent normal lead level. Her mother expressed concern for general parasitosis in their home community in Peru; however, the patient had never had specific symptoms and had not had any testing done.

In clinic, her vital signs were notable for tachycardia and tachypnea. She was afebrile. Her weight was in the 73rd percentile. Her general exam was notable for an uncomfortable appearance, not moving on the exam table. Her abdominal exam demonstrated active bowel sounds. She voluntarily tensed her abdominal muscles throughout the exam. She exhibited acute tenderness to palpation with rebound tenderness in the right lower quadrant (McBurney’s point) with generalized tenderness in all other quadrants, including the suprapubic region. Pain could be reliably elicited in her right lower quadrant with palpation of her left lower quadrant (Rovsing sign). Supine internal and external flexed hip rotation (obturator sign) and straight leg left against resistance (psoas sign) elicited intense pain in her right lower quadrant. She was unable to participate in a jump test to further assess for peritonitis.

Initial workup included a complete blood count (CBC) that showed a normal white blood cell count without anemia or platelet derangements but with a small elevation in eosinophils. While possibly incidental, the elevation was notable given her recent immigration, lack of prior testing or prophylactic treatment, and mother’s concern for parasitosis. An abdominal ultrasound was unable to visualize the appendix. Urinalysis showed elevated ketones but was otherwise unremarkable. Based on the elicited history, a stool ova and parasite test was sent and the patient was discharged home with strict return precautions. This course of action was taken as she was low risk for appendicitis based on the Pediatric Appendicitis Risk Calculator (pARC), her pain improved with oral acetaminophen, and the family lived near the hospital and had the ability to return quickly if needed. No abdominal computed tomography (CT) was ordered in favor of watchful waiting. Her pARC score of 14% was based on her sex, age, white blood cell count and neutrophilic percentage, duration of symptoms, maximal tenderness in her right lower abdominal quadrant, positive guarding without pain migration from the periumbilical region or demonstrated pain with walking. The following day, the stool ova and parasite test resulted positive for *Dientamoeba fragilis*. The patient’s family was contacted, and due to her persistent pain, she was prescribed metronidazole 3 times daily for 10 days with appropriate resolution of her symptoms.

## Discussion

To date, there is only 1 reported case of *D. fragilis* mimicking acute appendicitis.^7^ Additionally, literature suggests that parasitic involvement in appendicitis is often under-recognized—detected in only about 1.2% of cases—and the link to true inflammation remains debatable, highlighting significant gaps in epidemiological understanding and diagnostic protocols.^8^ Our case highlights 2 practical considerations in the workup of acute appendicitis: that in equivocal cases, *D. fragilis* should be considered, and more broadly, with the rise in global migration and the volume of newcomer children establishing care in the United States,^9^ parasitic infections should not be overlooked.

### D. fragilis as an Appendicitis Mimicker

The role of *D. fragilis* in acute and chronic abdominal pain is likely underappreciated. An intestinal protozoan, it preferentially inhabits the cecum and ascending colon. It has a global distribution with a greater prevalence in Europe and the Americas.^10^ The prevalence has been shown to be highly variable, however, and is dependent on the individual study populations and detection methods with measurements between 0.4% and 71% of specific populations.^11,12^ The mechanism by which *D. fragilis* is transmitted has not been conclusively elucidated, although the most accepted route is fecal-oral, and asymptomatic colonization rates in children can be high. It has been postulated that the nematode *Enterobius vermicularis* could serve as a vector. Both theories would also explain why *D. fragilis* has a higher affinity for children in daycare settings and has shown higher prevalence in populations experiencing limited sanitation or overcrowded conditions.^13^

In children, *D. fragilis* has been identified as benign, causing asymptomatic colonization, and treatment of asymptomatic infections is not indicated. Treatment is often reserved to patients with immunodeficiencies (usually hyper-IgM syndrome) or infections with severe symptoms in healthy children. However, there is a growing body of evidence that this protozoan could play a more active role in gastrointestinal disturbances than previously thought, since it is often not tested, and despite its known pathogenicity with well-established roles in both acute and chronic diarrhea and abdominal pain, it remains relatively unknown among general and subspecialized pediatricians.^14,15^ Moreover, it likely goes undetected in equivocal appendicitis cases as stool ova and parasite testing is not frequently obtained, suggesting that its causative role in abdominal pain could be greater than currently appreciated.^16^ In our case, with highly concerning exam findings but equivocal vital sign and laboratory findings of acute appendicitis, careful history taking not only led to a diagnosis but also spared the patient from further CT imaging—a relevant concern, with well-established links between childhood radiation exposure and future malignancies.^17,18^ Taken together, a nuanced history and thorough evaluation of confounding causes of acute appendicitis is critically important, with particular respect to *D. fragilis*.

### The Case for More Awareness of Parasitic Appendicitis Mimickers

This case sheds light on the complexities involved in providing care for newcomer children, a vulnerable population. *D. fragilis* is not the only pathogen to mimic acute appendicitis, many of which are more common in regions from which newcomer children are emigrating. Recent data from 2022 showed that 50% of immigration to the United States came from North America (Mexico), Central America, and South America.^19^ These populations have historically been underrepresented with respect to communicable disease outreach and there has been a growing focus on standardized newcomer healthcare strategies to systematically screen for common bacterial and viral pathogens. However, uniform parasitic testing has lagged behind and has mostly focused on *Strongyloides stercoralis* and schistosomiasis, neglecting the wide range of other parasites that are known to be overrepresented in these populations.^20^ Recent data from Spain showed that 23% of children who immigrated from Latin America were found to have symptomatic parasitic infections, the largest proportion being *Toxocara canis* and *Giardia intestinalis*, both of which can cause symptoms similar to appendicitis.^21^ Put in perspective, in an effort to reduce negative laparotomy rates, a recent retrospective analysis from Turkey attempted to assess the predictability of parasitic infection in patients with a clinical diagnosis of acute appendicitis. It found that 98% of the appendectomy samples from patients with clinical concern for parasitosis had histologic evidence of *E. vermicularis*, and almost all of those samples did not have histologically diagnostic appendicitis, suggesting that parasitosis can be a potent appendicitis mimicker.^22^ For patients from more rural areas and patients in high-density living environments often experienced by newcomer populations, these findings underscore the importance of expanded parasitological testing in the workup of acute appendicitis.

This approach should only apply in equivocal presentations and not in patients who meet a high index of suspicion for acute appendicitis; in addition, a normal eosinophil count should not rule out parasite infection, as *D. fragilis* itself only shows eosinophil elevation in 50% of cases.^23^ Care should also be given to this diagnostic approach, as *D. fragilis* colonization can be present with an unrelated appendicitis. Notably, when risk stratifying appendicitis, pARC has been validated in multiple prospective studies across pediatric and community emergency departments, showing high diagnostic accuracy with an area under the curve (AUC) of 0.89 (95% confidence interval 0.87–0.92), 100% sensitivity and 98.6% negative predictive value in very low-risk groups, and 99.0% specificity in high-risk groups.^24^ Compared to the older Pediatric Appendicitis Score (PAS), pARC offers superior performance, providing continuous risk estimates and higher AUCs in both community (0.89 vs. 0.80) and pediatric (0.85 vs. 0.77) settings. It also classifies more patients into actionable risk categories, improving clinical utility and reducing unnecessary interventions.^25^

Stool ova and parasite testing, while usually inexpensive and not difficult to interpret, can take time to provide results, which is a significant limitation when evaluating for acute abdomen. Nevertheless, this testing can be a valuable tool in situations when a patient can be discharged home with return precautions but should not supplant inpatient management if a higher level of care is indicated. Overall, this case is a salient reminder to maintain a broad differential that includes parasitic causes of abdominal pain in cases of suspected but equivocal appendicitis, particularly in vulnerable newcomer populations.

## Author Contributions

Dr Jason T. Tsichlis conceptualized the case report, drafted the initial manuscript, and critically reviewed and revised the manuscript. Dr Gaspar Rivera aided in defining the initial focus of the case report and critically reviewed and revised the manuscript. Both authors approved the final manuscript as submitted and agree to be accountable for all aspects of the work.

## References

[1] Goyal MK Chamberlain JM Webb M , et al. Racial and ethnic disparities in the delayed diagnosis of appendicitis among children. Acad Emerg Med. 2021;28(9):949-956. 32991770 10.1111/acem.14142

[2] Kharbanda AB Vazquez-Benitez G Ballard DW , et al. Development and validation of a novel pediatric appendicitis risk calculator (pARC). Pediatrics. 2018;141(4):e20172699. 10.1542/peds.2017-2699 PMC5869337 29535251

[3] Eslahi AV Olfatifar M Houshmand E , et al. Parasites in surgically removed appendices as a neglected public health concern: a systematic review and meta-analysis. Pathog Glob Health. 2022;116(6):341-355. 34842078 10.1080/20477724.2021.2008701 PMC9387334

[4] Taghipour A Olfatifar M Javanmard E Norouzi M Mirjalali H Zali MR. The neglected role of Enterobius vermicularis in appendicitis: a systematic review and meta-analysis. PLoS ONE. 2020;15(4):e0232143. 10.1371/journal.pone.0232143 PMC7179856 32324817

[5] Wylie L Van Meyel R Harder H , et al. Assessing trauma in a transcultural context: challenges in mental health care with immigrants and refugees. Public Health Rev. 2018;39:22-29. 30151315 10.1186/s40985-018-0102-y PMC6103972

[6] von Both U . Children on the move—a call for active screening in migrants. Lancet Child Adolesc Health. 2020;4(3):174-175. 31981478 10.1016/S2352-4642(19)30406-7

[7] Schwartz MD Nelson ME. Dientamoeba fragilis infection presenting to the emergency department as acute appendicitis. J Emerg Med. 2003;25(1):17-21. 12865103 10.1016/s0736-4679(03)00104-5

[8] Laor B Hassan AS. Parasitic appendicitis, what do we know? a literature review. Transl Gastroenterol Hepatol. 2025;10:32. doi:10.21037/tgh-24-148. 40337773 PMC12056108

[9] Abudiab S de Acosta D Shafaq S , et al. “Beyond just the four walls of the clinic”: the roles of health systems caring for refugee, immigrant and migrant communities in the United States. Front Public Health. 2023;11:1078980. 37064664 10.3389/fpubh.2023.1078980 PMC10097984

[10] Barry MA Weatherhead JE Hotez PJ Woc-Colburn L. Childhood parasitic infections endemic to the United States. Pediatr Clin North Am. 2013;60(2):471-485. 23481112 10.1016/j.pcl.2012.12.011

[11] Röser D Simonsen J Nielsen HV Stensvold CR Mølbak K. Dientamoeba fragilis in Denmark: epidemiological experience derived from four years of routine real-time PCR. Eur J Clin Microbiol Infect Dis. 2013;32(10):1303-1310. 23609513 10.1007/s10096-013-1880-2

[12] Wong ZW Faulder K Robinson JL. Does Dientamoeba fragilis cause diarrhea? a systematic review. Parasitol Res. 2018;117(4):971-980. 29404747 10.1007/s00436-018-5771-4

[13] Boga JA Rojo S Fernández J , et al. Is the treatment of Enterobius vermicularis co-infection necessary to eradicate Dientamoeba fragilis infection? Int J Infect Dis. 2016;49:59-61. 27263118 10.1016/j.ijid.2016.05.027

[14] Johnson EH Windsor JJ Clark CG. Emerging from obscurity: biological, clinical, and diagnostic aspects of Dientamoeba fragilis. Clin Microbiol Rev. 2004;17(3):553-570. 15258093 10.1128/CMR.17.3.553-570.2004 PMC452553

[15] Barratt JL Harkness J Marriott D Ellis JT Stark D. A review of Dientamoeba fragilis carriage in humans: several reasons why this organism should be considered in the diagnosis of gastrointestinal illness. Gut Microbes. 2011;2(1):3-12. 21637013 10.4161/gmic.2.1.14755

[16] Stark D Barratt J Chan D Ellis JT. Dientamoeba fragilis, the neglected trichomonad of the human bowel. Clin Microbiol Rev. 2016;29(3):553-580. 27170141 10.1128/CMR.00076-15 PMC4861990

[17] Berrington de Gonzalez A Pasqual E Veiga L. Epidemiological studies of CT scans and cancer risk: the state of the science. Br J Radiol. 2021;94(1126):20210471. 10.1259/bjr.20210471 PMC9328069 34545766

[18] Bosch de Basea Gomez M Thierry-Chef I Harbron R , et al. Risk of hematological malignancies from CT radiation exposure in children, adolescents and young adults. Nat Med. 2023;29(12):3111-3119. 37946058 10.1038/s41591-023-02620-0 PMC10719096

[19] Migration Policy Institute (MPI) tabulation of data from U.S. Census Bureau, 2010, 2019, and 2022 American Community Surveyes (ACS) and 2000 Decennial Census; data for 1960 to 1990 are from Campbell J. Gibson and Emily Lennon, “Historical Census Statistics on the Foreign-Born Population of the United States: 1850 to 1990” (Working paper No. 29, U.S. Census Bureau, Washington, DC, 1999).

[20] Norman FF Comeche B Chamorro S López-Vélez R. Overcoming challenges in the diagnosis and treatment of parasitic infectious diseases in migrants. Expert Rev Anti Infect Ther. 2020;18(2):127-143. 31914335 10.1080/14787210.2020.1713099

[21] Bustamante J Sainz T Ara-Montojo MF , et al. Screening for parasites in migrant children. Travel Med Infect Dis. 2022;47:102287. 35304329 10.1016/j.tmaid.2022.102287

[22] Zorlu M Şahiner İT Yastı AÇ , et al. Can clinical findings prevent negative laparotomy in parasitosis mimicking acute appendicitis? Adv Emerg Med. 2016;2016(1):8313604.

[23] Miguel L Salvador F Sulleiro E , et al. Clinical and epidemiological characteristics of patients with Dientamoeba fragilis infection. Am J Trop Med Hyg. 2018;99(5):1170-1173. 30328410 10.4269/ajtmh.18-0433 PMC6221219

[24] Cotton DM Vinson DR Vazquez-Benitez G , et al. Validation of the Pediatric Appendicitis Risk Calculator (pARC) in a community emergency department setting. Ann Emerg Med. 2019;74(4):471-480. doi:10.1016/j.annemergmed.2019.04.023. 31229394 PMC8364751

[25] American College of Emergency Physicians Clinical Policies Subcommittee (Writing Committee) on Appendicitis, Diercks DB Adkins EJ , et al. Clinical policy: critical issues in the evaluation and management of emergency department patients with suspected appendicitis: approved by ACEP Board of Directors February 1, 2023. Ann Emerg Med. 2023;81(6):e115-e152. doi:10.1016/j.annemergmed.2023.01.015. 37210169

